# Supplementary material for: β1-Integrin-Mediated Uptake of Chondrocyte Extracellular Vesicles Regulates Chondrocyte Homeostasis
Source: Int J Mol Sci. 2024 Apr 26;25(9):4756. doi: 10.3390/ijms25094756 (PMC11083596; doi:10.3390/ijms25094756)
Supplement: Supplementary file 1 [file ijms-25-04756-s001.zip › ijms-2919284-supplementary.pdf]

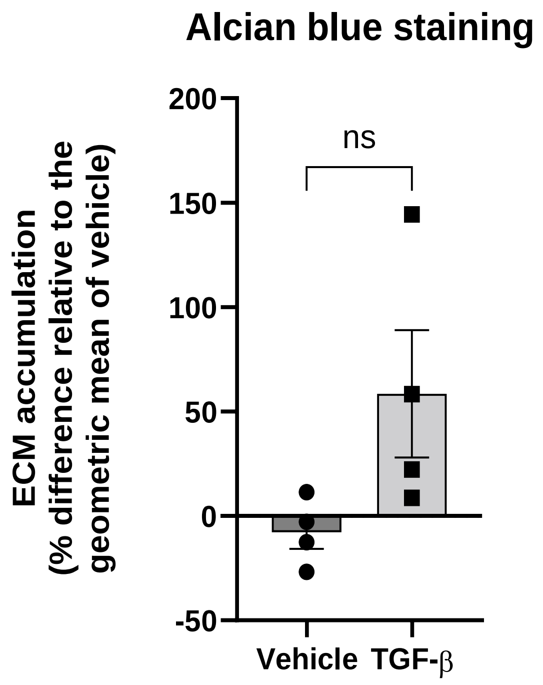

Figure S1. Alcian blue staining of 3D cultures treated with vehicle or TGF-β. Percentage of extracellular matrix accumulation represented by alcian blue staining normalised to DNA content. Staining was normalised to the geometric mean of vehicle treated cultures. Data represent the mean ± SEM (n=4; Unpaired T-test (ns= p>0.05).

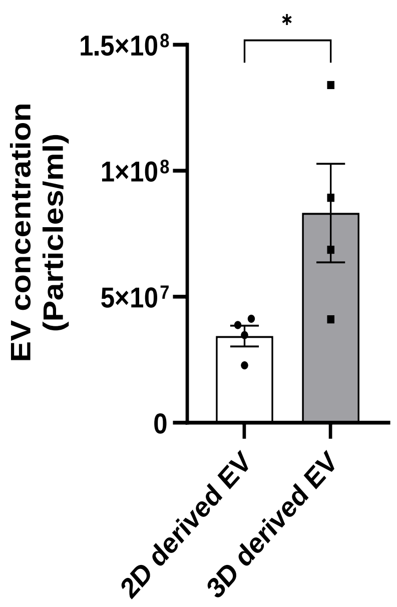

Figure S2. Response of chondrocytes to different chondrocyte EV preparations. Concentration of newly generated EVs isolated from chondrocyte monolayers 24h after treatment with EVs isolated from 2D or 3D chondrocyte cultures. Data represent the mean ± SEM (n=4; Unpaired T-Test (*= $p<0.05$).

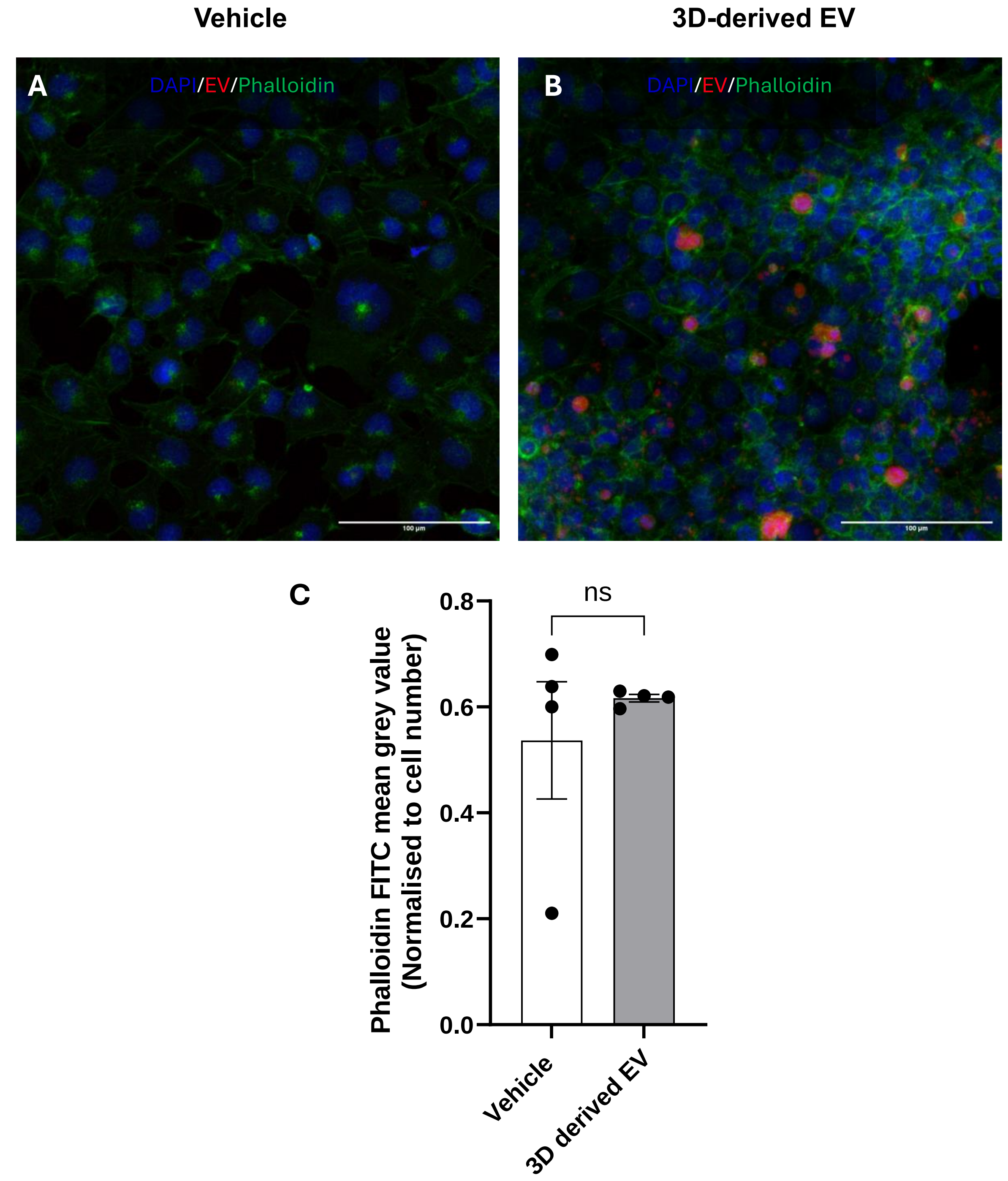

Figure S3. Phalloidin staining of chondrocytes following treatment with vehicle or 3D-derived EVs. Representative confocal images of chondrocytes treated with **A.** Vehicle or **B.** 3D-derived EVs (red), chondrocytes are stained with DAPI for the nucleus (blue) and cytoskeletal staining with phalloidin (green). **C.** Mean grey value of phalloidin staining normalised to cell number from cells treated with vehicle or 3D-derived EVs. Data represent the mean ± SEM (n=4; Unpaired T-test (ns= $p>0.05$).
